# Identification of Gemin5 as a Novel 7-Methylguanosine Cap-Binding Protein

**DOI:** 10.1371/journal.pone.0007030

**Published:** 2009-09-14

**Authors:** Shelton S. Bradrick, Matthias Gromeier

**Affiliations:** 1 Department of Molecular Genetics and Microbiology, Duke University Medical Center, Durham, North Carolina, United States of America; 2 Department of Surgery, Duke University Medical Center, Durham, North Carolina, United States of America; Institute of Molecular and Cell Biology, Singapore

## Abstract

**Background:**

A unique attribute of RNA molecules synthesized by RNA polymerase II is the presence of a 7-methylguanosine (m^7^G) cap structure added co-transcriptionally to the 5′ end. Through its association with trans-acting effector proteins, the m^7^G cap participates in multiple aspects of RNA metabolism including localization, translation and decay. However, at present relatively few eukaryotic proteins have been identified as factors capable of direct association with m^7^G.

**Methodology/Principal Findings:**

Employing an unbiased proteomic approach, we identified gemin5, a component of the survival of motor neuron (SMN) complex, as a factor capable of direct and specific interaction with the m^7^G cap. Gemin5 was readily purified by cap-affinity chromatography in contrast to other SMN complex proteins. Investigating the underlying basis for this observation, we found that purified gemin5 associates with m^7^G-linked sepharose in the absence of detectable eIF4E, and specifically crosslinks to radiolabeled cap structure after UV irradiation. Deletion analysis revealed that an intact set of WD repeat domains located in the N-terminal half of gemin5 are required for cap-binding. Moreover, using structural modeling and site-directed mutagenesis, we identified two proximal aromatic residues located within the WD repeat region that significantly impact m^7^G association.

**Conclusions/Significance:**

This study rigorously identifies gemin5 as a novel cap-binding protein and describes an unprecedented role for WD repeat domains in m^7^G recognition. The findings presented here will facilitate understanding of gemin5's role in the metabolism of non-coding snRNAs and perhaps other RNA pol II transcripts.

## Introduction

A salient feature of RNA transcripts produced by eukaryotic RNA polymerase II is the presence of a 7-methylguanosine (m^7^G) cap structure at the 5′ terminus. In metazoans a two-component enzyme complex engendering triphosphatase, guanylyltransferase, and methyltransferase enzymatic activities is responsible for modifying RNAs at the 5′ end with m^7^G [1]. These critical functions are conserved in fungi and the human capping system can replace that of *Saccharomyces cerevisiae* [2]. The nuclear capping reaction serves as an important checkpoint for RNA quality control. Multiple studies have documented that efficient pre-mRNA splicing and polyadenylation depend upon an intact 5′ cap [3], [4]. Furthermore, the cap structure is an important determinant of mRNA stability in both the nucleus and cytoplasm [5].

Functions attributed to the cap depend upon specific trans-acting factors that have been shown to directly bind m^7^G. In the nucleus a bipartite cap-binding complex (CBC) composed of CBP20 and CBP80 associates with nascent pre-mRNAs to promote splicing and export [6], [7]. After gaining access to the cytoplasm, mRNPs are remodeled to produce translation-competent particles. This process minimally involves exchange of the CBC for eukaryotic initiation factor (eIF) 4E, the predominantly cytoplasmic cap-binding protein responsible for directing the initiation phase of translation [8], [9]. Subsequent to translation, the cap plays an important role in determining the end of an mRNA's existence. Once cytoplasmic mRNA is deadenylated, irreversible decapping by DCP2 complexed with DCP1 results in rapid decay of the transcript body [10]. Thus, the m^7^G cap moiety participates in every major aspect of mRNA metabolism.

In addition to mRNAs, RNA pol II mediates the synthesis of multiple non-coding RNAs including the Sm class of small nuclear (sn) RNAs composed of eight unique species: U1, U2, U4, U4_atac_, U5, U7, U11, and U12 [11]. These snRNAs are also modified co-transcriptionally with a 5′ m^7^G cap, but 3′ end processing is carried out by distinct machinery that generates mature non-polyadenylated termini. Similar to mRNAs, newly synthesized Sm class snRNAs are bound by CBC which, along with the adaptor protein PHAX [12], facilitates export to the cytoplasm where snRNAs undergo further maturation.

Eukaryotes have evolved macromolecular protein complexes of varying complexity [13] that are responsible for cytoplasmic assembly of snRNPs. In humans the survival of motor neuron (SMN) complex mediates the assembly and specific deposition of heptameric Sm cores on the conserved Sm site (5′-AUUU/CUUG-3′) within snRNAs [14], [15], [16], [17]. In addition to SMN protein, the SMN complex is constituted by at least seven factors known as gemins. The largest component of the SMN complex is gemin5, a ∼170 kDa WD repeat-containing protein that is conserved among vertebrates [17], [18]. Like other SMN complex members, gemin5 is predominantly cytoplasmic but may also localize to distinct sub-nuclear foci known as gems or Cajal bodies [19]. Gemin5 has been implicated as the SMN constituent that confers specificity to the SMN complex for Sm class snRNA substrates [15]. This specificity depends upon recognition by gemin5 of the “snRNP code” which consists of the Sm site and at least one 3′ proximal stem-loop [20]. Interestingly, a significant fraction of cytoplasmic gemin5 appears to exist free of the SMN complex or in a sub-complex with gemin3 and gemin4 [21]. Gemin5 also uniquely dissociates from the SMN complex under conditions of high salinity [22]. Thus, gemin5 may be considered a peripheral component of the SMN complex.

Subsequent to Sm core assembly, the snRNA cap is hypermethylated by the TGS1 methyltransferase to form 2,2,7-trimethylguanosine (TMG) [23], [24]. The import factor snurportin directly recognizes the TMG cap [25] and, in cooperation with SMN [26], directs nuclear import of the assembled snRNP for final maturation steps before assembly into active spliceosomes. Hence, as with mRNA metabolism, the 5′ cap is essential for biogenesis of snRNAs.

While it is well established that capping of RNA pol II transcripts with m^7^G is critical for their metabolism, it is not clear whether the full complement of cellular proteins capable of binding the cap structure has been defined. Here, we present evidence that gemin5 is capable of direct and specific interaction with m^7^G in a manner that depends upon integrity of its WD repeat domains. These findings expand the repertoire of identified cellular m^7^G cap-binding proteins and raise the possibility that gemin5 is a novel regulator of gene expression at the post-transcriptional level.

## Results

### Gemin5 is specifically purified by cap-affinity chromatography

**Figure 1:**
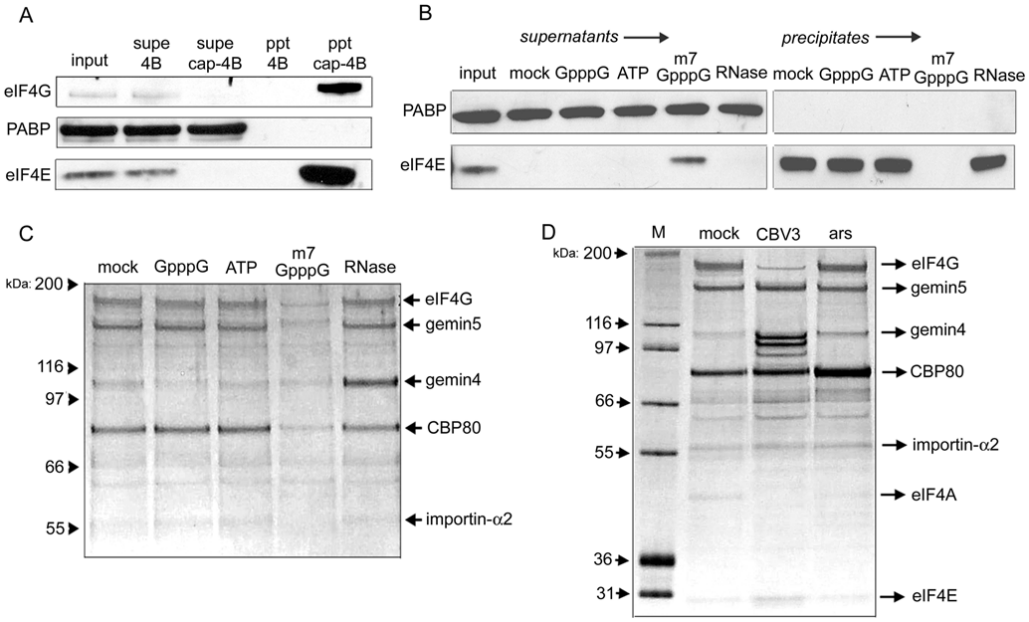
Characterization of proteins purified by cap-affinity chromatography. (A) HeLa cytoplasmic lysate was applied to m^7^G-sepharose 4B or sepharose 4B alone. Precipitates (ppt) were washed extensively and then resuspended in sample buffer. Levels of eIF4G, PABP and eIF4E in input and supernatant (supe) samples were examined by western blot. Input samples represent 10% of total. (B) Cap-affinity chromatography was performed in the presence of the indicated nucleotide (0.1 mM final concentration) or 0.1 mg/ml RNase A and PABP/eIF4E were detected by western blot. (C) The same precipitate samples shown in (B) were subjected to silver stain detection. The identities of proteins determined by mass spectrometry are indicated. (D) HeLa lysates were derived from normally growing cells (mock), cells infected with CBV3 at four hours post-infection, or cells acutely stressed with 0.1 mM arsenite for 30 minutes. Proteins precipitated with cap-sepharose were then analyzed by silver stain.

Efficient isolation of cap-binding complexes from cell lysates can be achieved using m^7^GTP immobilized to sepharose by covalent linkage [27]. Cap-sepharose is widely used to investigate interaction between eIF4E and its binding partners. Chief among these is the scaffolding protein eIF4G, which is believed to mediate ribosomal recruitment through interaction with eIF3 [28], [29]. Exposure of cytoplasmic HeLa cell lysate to cap-sepharose precipitates both eIF4E and eIF4G, in contrast to sepharose alone (Figure 1A). Under the binding conditions employed here (see experimental procedures), the abundant poly(A)-binding protein (PABP) was not detectable in cap-resin precipitates and thus served as an input control. Cap-affinity chromatography performed in the presence of various reagents (GpppG, ATP, m^7^GpppG, RNase A) indicated robust stringency of the purification method (Figure 1B). In particular, addition of free m^7^GpppG to binding reactions prevented eIF4E precipitation while unmethylated cap analog (GpppG) had no effect, demonstrating strict binding specificity of eIF4E to methylated guanosine. As with GpppG, supplementation of reactions with ATP or treatment of lysate with RNase did not affect eIF4E precipitation (Figure 1B).

We questioned whether proteins besides eIF4E and eIF4G were specifically purified by cap-affinity. Analysis of precipitates shown in figure 1B by silver stain indicated the presence of five proteins migrating between 55 and 200 kDa whose abundances were specifically reduced by free m^7^GpppG (Figure 1C). The quantity of a ∼100 kDa protein increased substantially as a consequence of RNase treatment, suggesting that disruption of endogenous RNPs allows more efficient purification of this factor. These proteins were subsequently isolated in a separate purification and analyzed by mass spectrometry. This analysis revealed the identities of these proteins as importin-α, CBP80, gemin4, gemin5, and eIF4G (Figure 1C). CBP80 and eIF4G were expected constituents of cap-binding complexes. Additionally, importin-α is a known interaction partner of the CBC that mediates its nuclear import subsequent to release of the CBC's RNA cargo [30]. However, the presence of SMN complex components, gemin4 and gemin5, was unexpected.

The pattern of proteins purified by cap-affinity was also examined using lysates from cells acutely stressed with arsenite or infected with coxsackievirus B3 (CBV3). CBV3 encodes a protease (2A^pro^) that cleaves eIF4G downstream of the eIF4E interaction site [31]. This resulted in markedly reduced purification of intact eIF4G and corresponding precipitation of ∼100 kDa N-terminal fragments that retain the ability to interact with eIF4E (Figure 1D). A protein band corresponding in size to eIF4A was also expectedly absent, since the C-terminal eIF4G fragment produced by 2A^pro^ harbors eIF4A-binding sites [28], [32]. Isolation of other factors was unaffected by CBV3 infection. Short-term arsenite exposure to induce oxidative stress did not modify the pattern of protein pull-down, but appeared to result in elevated CBP80 purification (Figure 1D).

**Figure 2:**
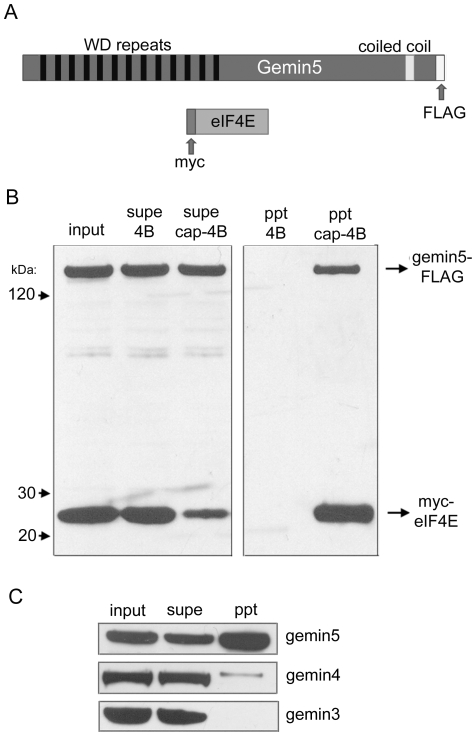
Gemin5 isolated by cap-affinity is not bound to the SMN complex. (A) Schematic representation of gemin5 and eIF4E indicating locations of WD repeats, the putative coiled coil domain, and epitope tags. (B) 293T cells were co-transfected with expression constructs encoding FLAG-tagged gemin5 and myc-tagged eIF4E. Cell lysate was derived 24 hours later and applied to cap-sepharose 4B or sepharose 4B. Tagged proteins were detected with α-FLAG and α-myc antibodies. (C) Purification of proteins by cap-affinity was performed as in figure 1A and levels of endogenous gemin3–5 were examined in input, supernatant and precipitate samples.

Since RNase treatment significantly enhanced gemin4 recovery by cap-affinity without affecting gemin5 pull-down, we reasoned that gemin4 isolation was secondary to its binding partner gemin5, and thus focused our study on the latter. In order to confirm mass spectrometric findings, we generated cDNA clones for transient over-expression of tagged gemin5 and eIF4E proteins (Figure 2A). As with the corresponding endogenous factors, C-terminally FLAG-tagged gemin5 and N-terminally myc-tagged eIF4E co-expressed in 293T cells both specifically precipitated on cap-sepharose but not sepharose alone (Figure 2B). We also examined the levels of endogenous gemin5 and its binding partners gemin3 and gemin4 in cap-affinity chromatography by western blot (Figure 2C). As expected, endogenous gemin5 was present in precipitates, but comparatively little gemin4 was purified and gemin3 was undetectable. This suggests that the predominant form of gemin5 purified by cap-affinity is not bound to gemin3/4 or the SMN complex. Interestingly, we consistently observed that only a fraction of endogenous or over-expressed gemin5 was precipitated with cap-resin (Figures 2B and2C; compare levels in “input” and “supe” lanes), whereas eIF4E was efficiently depleted from lysates (Figures 1A, 1B and 2B).

### The WD repeat domains of gemin5 are required for association with cap-binding complexes

**Figure 3:**
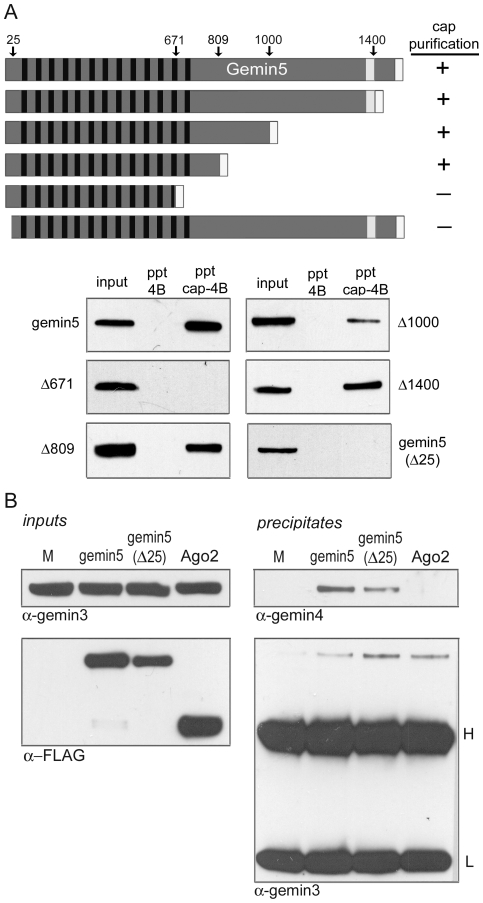
Mapping of determinants within gemin5 required for association with m^7^G-sepharose. (A) Multiple C-terminal truncations and a single N-terminal truncation of FLAG-tagged gemin5 were evaluated by cap-affinity chromatography. Deletion sites within gemin5 are indicated above by amino acid number. (B) FLAG-tagged gemin5, gemin5(Δ25) variant, and Ago2 expression constructs were transfected into 293T cells along with pcDNA3 alone (M). Input samples used for co-IP assays were analyzed by detection of gemin3 and over-expressed FLAG-tagged proteins (left). Each lysate was subjected to IP using α-FLAG antibody and precipitate samples were analyzed for the presence of gemin3 and gemin4. Positions of heavy (H) and light (L) antibody chains are indicated.

We set out to identify region(s) of gemin5 that are required for association with cap-sepharose by introducing serial deletions into the FLAG-tagged gemin5 cDNA construct. Gemin5 is 1508 amino acids in length and contains 13 WD repeats in its N-terminal half. Characteristic β-propeller structures are known to be formed by 6 to 7 WD repeat domains [33], suggesting that gemin5 contains two adjacent β-propellers (see below). Deletions of 108, 508, or 699 amino acids from the C-terminus had no effect on isolation by cap-affinity (Figure 3A). However, truncation of 837 residues, resulting in disruption of two intact WD repeats, completely abrogated binding to cap-sepharose. We also performed deletion analysis of the authentic gemin5 N-terminus. Surprisingly, a minimal deletion of 25 amino acids abolished association with the cap-resin (Figure 3A). Although these N-terminal residues precede the first WD repeat, which begins at amino acid 57, structural modeling suggests that they may participate in one of gemin5's β-propellers (see discussion).

We next questioned whether the minimal N-terminal deletion, hereafter referred to as gemin5(Δ25), affected interaction with binding partners gemin3 and gemin4. 293T cells were transfected with plasmids expressing tagged gemin5, gemin5(Δ25), or vector DNA. For additional comparison, we also expressed an N-terminally FLAG-tagged version of human argonaute2 (Ago2), a component of the RNA-induced silencing complex (RISC) that has been reported to interact with gemin3 and gemin4 [34], [35]. Levels of gemin3 were monitored by western blot to ensure equal protein content in “input” samples (Figure 3B). Immunoprecipitations (IPs) using α-FLAG antibody were performed on each lysate and precipitates were probed for the presence of gemin3 and gemin4. Both intact gemin5 and the gemin5(Δ25) mutant specifically co-immunoprecipitated gemin3 and gemin4 while Ago2 appeared to co-purify only gemin3 (Figure 3B). Thus, the inability of gemin5(Δ25) to bind cap-sepharose does not correlate with loss of gemin3 or gemin4 interaction.

### Gemin5 is an authentic cap-binding protein

**Figure 4:**
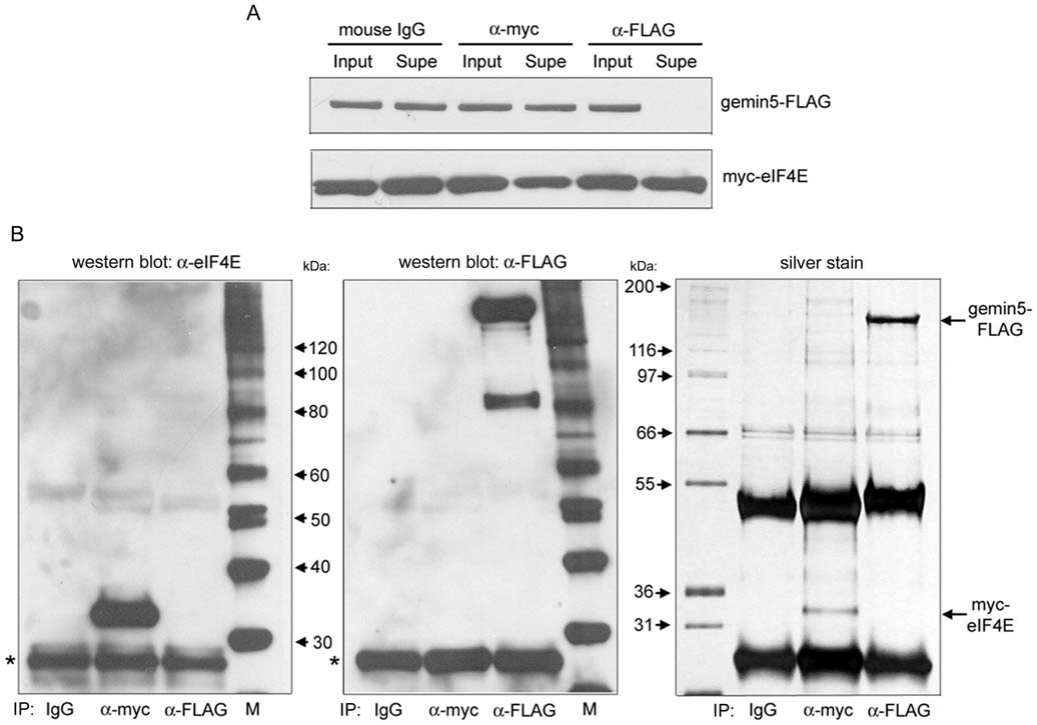
Gemin5 fails to detectably interact with eIF4E in co-IP experiments. 293T cells were co-transfected with gemin5 and eIF4E expression constructs as in figure 2 and cytoplasmic lysate was used for IP using the indicated murine antibodies (IgG, α-myc, and α-FLAG). (A) Input and supernatant samples assayed for levels of over-expressed proteins. (B) Each IP was analyzed by western blot with α-eIF4E (left) and α-FLAG (middle) rabbit antibodies, and by silver stain (right). Blots were intentionally over-exposed to assess possible co-IP. Asterisks indicate mouse light antibody chain used in IP reactions that cross reacts with rabbit secondary antibody.

We sought to identify the molecular basis underlying the observation that gemin5 binds to m^7^G cap-sepharose. One possibility is that gemin5 binds to the prototype cap-binding protein, eIF4E. Indeed, gemin5 has been recently suggested to be a novel eIF4E-interaction partner (Fierro-Monti et al., 2006). To investigate this possibility we co-expressed tagged gemin5 and eIF4E in 293T cells and performed co-IP assays using pre-immune, α-myc tag, and α-FLAG tag antibodies (Figure 4A and B). Each antibody clearly and specifically precipitated its target protein as evidenced by both western blot and silver stain. However, there was no detectable co-IP of either protein suggesting that eIF4E and gemin5 do not form a stable complex in 293T cells.

**Figure 5:**
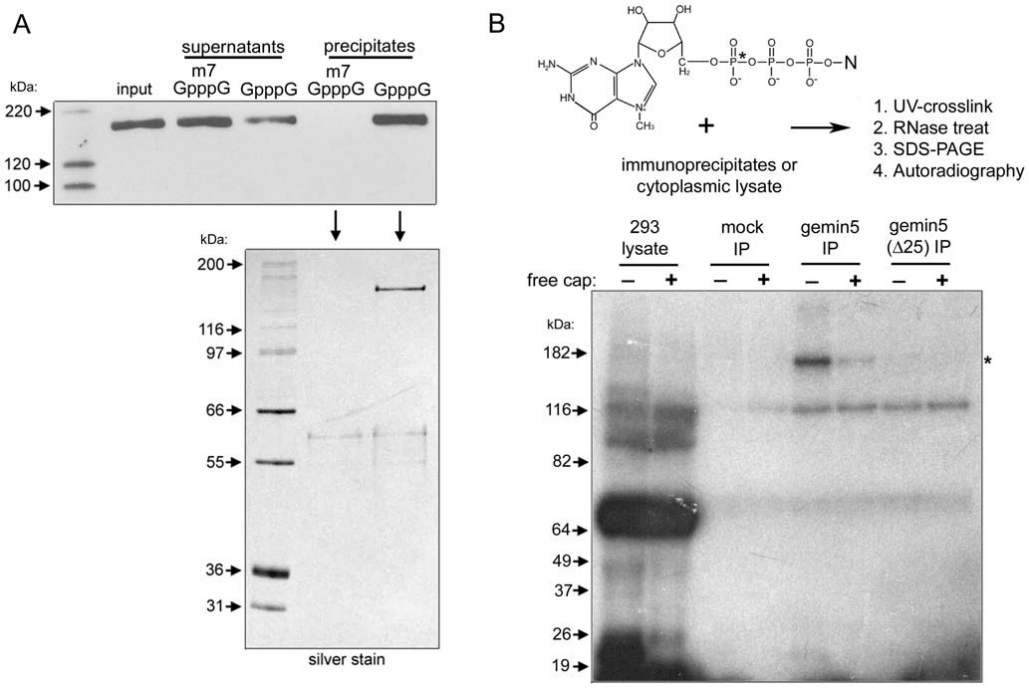
Gemin5 binds directly to the m^7^G cap structure. (A) FLAG-tagged gemin5 was immunoprecipitated and released from the resin with FLAG peptide. Eluted protein was subsequently applied to cap-sepharose in the presence of free m^7^GpppG or GpppG competitors. Precipitation of FLAG-gemin5 was monitored by western blot and silver stain analysis. (B) A representation of the m^7^G cap structure is shown with position of radiolabeled α-phosphate indicated by an asterisk. A short RNA transcript was synthesized and then modified with an m^7^G cap using [α-^32^P]-GTP and guanylyltransferase (see experimental procedures for details). Purified capped RNA was incubated in the presence or absence 0.1 mM free cap analog with 1) 293T cytoplasmic lysate, 2) eluate from α-FLAG IP of lysate from pcDNA3-transfected cells (mock IP), or 3) FLAG-tagged gemin5 or gemin5(Δ25) immunopurified as in (A). Binding reactions were irradiated with UV light, incubated with RNase cocktail, and then analyzed by SDS-PAGE followed by autoradiography as indicated.

Immunoprecipitated FLAG-tagged proteins can be efficiently eluted under native conditions using FLAG peptide. We used this approach to purify gemin5 and perform subsequent cap-affinity chromatography in the presence of specific (m^7^GpppG) or nonspecific (GpppG) cap analog competitors (Figure 5A). Remarkably, gemin5 was precipitated on cap-sepharose exclusively in the presence of the nonspecific GpppG competitor. This observation indicates that eIF4E is not responsible for tethering gemin5 to m^7^G. Silver stain analysis of precipitates from these binding reactions revealed the presence of gemin5, but other specific co-precipitating proteins were undetectable (Figure 5A). This result suggested that gemin5 itself is capable of direct and specific interaction with the m^7^G cap structure.

In order to rigorously test this possibility, we employed a strategy based on UV-induced crosslinking of proteins directly to radiolabeled cap structure (Figure 5B). An in vitro transcribed RNA was capped with guanylyltransferase using [α-^32^P]GTP and then incubated with immunopurified intact gemin5 or the gemin5(Δ25) deletion mutant in the presence or absence of free cap analog competitor. As an additional control, eluate from FLAG-IP of mock-transfected cells was also used. After incubation, reactions were irradiated with UV light, treated with an RNase cocktail, and then subjected to SDS-PAGE. In reactions using total cytoplasmic lysate, most crosslinked proteins bound the capped RNA nonspecifically since addition of free cap analog had no effect on their crosslinking efficiency (Figure 5B). Importantly, purified intact gemin5 was strongly crosslinked in a manner that was specifically negated by the presence of free cap structure. Moreover, the gemin5(Δ25) mutant was not specifically labeled by the capped RNA. Together with data presented in figure 5A, these results reveal that gemin5 is a protein with m^7^G cap-binding capability.

### Identification of amino acid residues that mediate gemin5 binding to m^7^G

**Figure 6:**
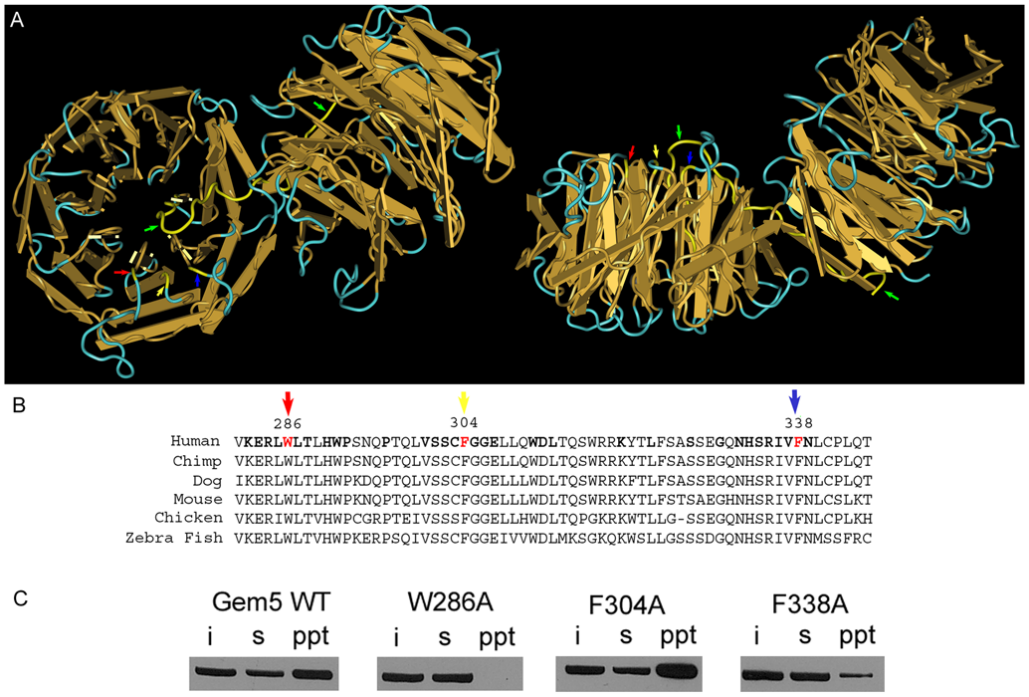
Identification of amino acid residues that affect m^7^G recognition by gemin5. (A) PHYRE analysis was used to model the gemin5 WD repeat domains onto the structure of actin-interacting protein 1 (AIP-1; see Materials and Methods). The backbone of the AIP-1 structure, consisting of two β-propellers, is shown with indicated locations of amino acids highlighted in yellow. A 90° x-axis rotation of the left structure is shown at right. Arrows indicate positions of W286 (red), F304 (yellow), F338 (blue) and the N-terminal 25 amino acids of AIP-1 (green). Note that the N-terminus of AIP-1 forms a β-sheet in the last WD repeat domain of the second β-propeller before looping into the first β-propeller to form another β-sheet. PHYRE analysis predicts only the second β-sheet in gemin5. AIP-1 structures were visualized using Cn3D [59]. (B) Alignments of gemin5 sequences from selected vertebrate species is shown. Bold letters in the human sequence indicate uniform conservation and positions of residues 286, 304 and 338 are indicated. (C) Cap-binding assays were performed with wild-type gemin5-FLAG and variants as in previous figures. Input (i), supernatant (s), and precipitate (ppt) samples are indicated.

Recently, Lau et al. mapped the binding site for U4 snRNA on gemin5 in vitro [36]. These authors found that disruption of the WD repeat domains abrogated U4 snRNA binding whereas the C-terminal half of gemin5 was largely dispensable. RNA-mediated hydroxyl radical probing further revealed that U4 snRNA contacts gemin5 near W286, located at the beginning of the fifth WD repeat. Lau et al. performed structural modeling using the Protein Homology/analogy Recognition Engine (PHYRE)[37] which allowed threading of the gemin5 WD repeat domains onto the known structure of actin-interacting protein 1 (AIP-1) [38]. This analysis predicted W286 to be solvent exposed and located on the surface of the first β-propeller (Figure 6A). Moreover, mutation of this amino acid to alanine (W286A) significantly disrupted U4 snRNA-gemin5 interaction.

Since we also found that gemin5's m^7^G-binding capability maps to the WD repeat domains, we tested whether the W286A mutant might affect cap-binding. FLAG-tagged gemin5 and the W286A mutant were transiently expressed in 293T cells and cap pull-down assays were performed as in previous figures. Strikingly, mutation of amino acid 286 abolished association with cap sepharose (Figure 6C), indicating that this tryptophan is critical for interaction with both m^7^G and U4 snRNA.

Structural characterizations of several viral and eukaryotic cap-binding proteins have demonstrated that association with m^7^G significantly depends upon aromatic residues that stack on either side of the guanine base (see discussion). Since W286 is critical for gemin5's ability to recognize m^7^G, we hypothesized that a second aromatic residue within the vicinity of position 286 might participate in cap-binding. Similarly to Lau et al., we utilized PHYRE to identify predicted locations of aromatic residues that are both solvent-exposed and located within 8 Å of W286. This distance was selected because the solved structures of eIF4E and CBP20 reveal interplanar distances between m^7^G and aromatic side chains to be ∼3.5 Å. Two amino acids fit these criteria, F304 and F338, with predicted distances from W286 of approximately 3 and 6 Å, respectively (Figure 6A). Each of these residues is located on the exposed surface of one side of the β-propeller adjacent to W286. In addition, all three of these amino acids are conserved in vertebrate homologs of gemin5 (Figure 6B).

We established mutant, FLAG-tagged gemin5 expression constructs F304A and F338A and tested their cap-binding activity side by side with wild-type gemin5 and W286A (Figure 6C). Mutation of F304 did not affect purification by cap-affinity chromatography. In contrast, the F338A variant exhibited significantly reduced association with cap-sepharose compared to either wild-type gemin5 or the F304A construct but, unlike W286A, was still detectable in cap precipitates. These findings indicate that F338 and W286 are important for cap recognition by gemin5 and suggest these residues may participate in stacking interactions with m^7^G as in structurally-characterized cap-binding proteins. On the other hand, we cannot rule out the possibility that W286A and/or F338A mutations affect global structure of gemin5 in a manner that reduces affinity for the m^7^G cap.

### Analysis of gemin5 association with endogenous U1 snRNA

**Figure 7:**
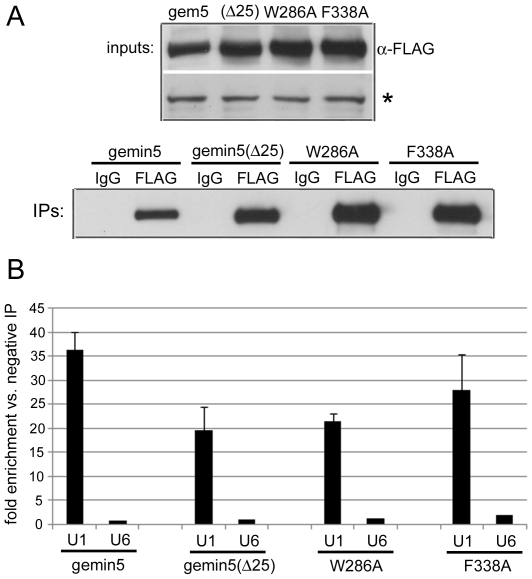
Gemin5 mutations that affect m^7^G interaction reduce association with U1 snRNA. (A) Gemin5-FLAG and the indicated variants were transiently expressed in 293T cells and then immunoprecipitated with α-FLAG antibody or negative control mouse IgG. A 10% fraction of IP samples along with input samples were subjected to α-FLAG western blot. The asterisk indicates a cross-reactive band that serves as a loading control. The remaining IPs were used for RNA extraction. (B) RT-qPCR was performed on extracted RNAs for measurements of U1 and U6 snRNA levels in positive and negative IP samples for each gemin5 variant. Error bars indicate values for standard deviation.

We next examined the effects of gemin5 mutations on association with U1 snRNA, a well-known substrate of the SMN complex. RNA immunoprecipitation (RIP) and quantitative RT-PCR (RT-qPCR) methods were employed to measure levels of endogenous U1 snRNA co-precipitating with FLAG-tagged gemin5 over-expressed in 293T cells (Figure 7). The same analysis was applied simultaneously to gemin5(Δ25) and the W286A/F338A amino acid substitution mutants that display significantly reduced binding to m^7^G. Compared to negative control RIP with species-matched IgG, gemin5 RIP enriched U1 snRNA by approximately 35-fold (Figure 7B). This was specific for U1 snRNA as measurements of the Lsm class U6 snRNA, a non-substrate of the SMN complex, were equivalent in gemin5 and negative control RNA immunoprecipitates. Interestingly, each mutant version of gemin5 specifically enriched U1 snRNA compared to negative controls, perhaps reflecting a degree of indirect association of gemin5 with U1 snRNA through other SMN complex components. Nevertheless, each mutant consistently co-precipitated U1 snRNA to a lesser extent than intact, wild-type gemin5 (Figure 7B) despite somewhat less abundant expression and immunoprecipitation of the latter (Figure 7A). Taken together with data presented in figure 6, these observations along with those made by Lau et al. suggest that determinants of binding to m^7^G and U1 snRNA are closely associated in the WD repeat domains of gemin5.

## Discussion

Cellular proteins capable of recognizing the m^7^G cap-structure added co-transcriptionally to all RNA pol II transcripts execute critical functions in mRNA and snRNA metabolism. Here, we identify gemin5, a peripheral component of the SMN complex, as a novel m^7^G cap-binding protein. Gemin5 was specifically purified by cap-affinity chromatography (Figures 1 and 2), but did not detectably interact in co-IP assays with the prototypic cap-binding protein, eIF4E (Figure 4). Crucially, immunopurified gemin5 bound cap-sepharose in the absence of other proteins, and specifically crosslinked to radiolabeled cap-structure after UV irradiation (Figure 5). The latter being the most stringent criterion for evaluating putative cap-binding activity [6], [8], [39]. Thus, gemin5 may be considered a new member of the m^7^G cap-binding class of proteins.

Despite lack of sequence homology, biophysical investigations have revealed a similar structural motif involved in cap recognition shared among multiple identified cellular and viral cap-binding proteins including eIF4E [40], CBP20 [41], 4E-HP [42], vaccinia virus VP39 [43], and influenza virus PB2 [44]. Each of these proteins bind the cap structure via conserved aromatic side chains that stack on either side of m^7^G, forming a cap “sandwich”. We investigated the molecular determinants responsible for gemin5 interaction with m^7^G. Deletion analysis revealed that integrity of the 13 WD repeats present in the N-terminal half of gemin5 are important for cap-binding, indicating an unprecedented structural association between the propeller-like platforms known to be formed by tandemly-arrayed WD repeats and the cap-binding motif (Figure 3). Moreover, structural modeling using the PHYRE algorithm combined with site-directed mutagenesis putatively identified unique aromatic amino acids (W286 and F338; located in the 5^th^ and 6^th^ WD repeats, respectively) that may directly mediate cap interaction similar to previously characterized cap-binding proteins (Figure 6). However, the possibility that gemin5 associates with the cap structure via an alternative mode cannot be ruled out. For example, poly(A)-specific ribonuclease (PARN) has been demonstrated to bind m^7^G via a single one-sided stacking interaction involving tryptophan [45], [46], [47]. The PHYRE analysis also suggested an integral role for involvement of the N-terminal 25 residues in the structure of the first β-propeller. In particular, this region is predicted to compose the first β-strand of the N-terminal WD repeat (Figure 6A), and deletion of these amino acids may have broad destabilizing effects that preclude specific recognition of m^7^G.

In contrast to eIF4E, cap-affinity chromatography consistently failed to significantly deplete gemin5 from cell lysates (Figures 1 and 2). One possible explanation for this finding is that the steady-state affinity of gemin5 for the cap is significantly lower compared to that of eIF4E. On the other hand, gemin5's cap-binding capability may be negatively regulated by interaction with its partner protein(s). Notably, gemin5 purified on cap-sepharose minimally co-precipitates gemin4 without RNase treatment and fails to pull down gemin3, although direct IP of gemin5 efficiently co-purifies both proteins [21](our unpublished data). These observations indicate that gemin5 purified by cap-affinity is largely a free subunit not bound to gemin3/4 or the entire SMN complex.

Gemin5 has been implicated as the factor responsible for identifying known substrates of the SMN complex, the Sm-class snRNAs. Previous observations indicate that an intact “snRNP code”, generically consisting of the Sm site and a 3′ proximal stem-loop, is necessary and sufficient for recognition of Sm class snRNAs by gemin5 [36], [48]. Thus, although Sm-class snRNAs are capped during transcription by RNA pol II, the m^7^G moiety is apparently dispensable for specific binding to gemin5, at least in vitro. Interestingly, W286 is required for maximum gemin5 binding to both m^7^G and U1/U4 snRNAs (Figures 6 and 7) [36], indicating that the recognition site(s) for these ligands at least partially overlap. It will be of interest to determine whether cap- and snRNA-binding to gemin5 are mutually exclusive.

Gemin5 has been reported to accumulate in stress granules [21], discrete cytoplasmic foci that contain specific initiation factors, RNA-binding proteins, small (40S) ribosomal subunits and translationally-silent mRNAs [49]. Since gemin5 is also capable of cap-binding, it may conceivably participate in RNP complexes containing mRNAs. Hypothetical gemin5 mRNPs would likely exist in translationally-silent, sub-polysomal complexes through preclusion of eIF4E association with the cap. Indeed, the vast majority of gemin5, whether as free protein or bound to the SMN complex, is present in translationally inactive fractions of polysome gradients [21](our unpublished data). Notably, gemin5 has been recently reported to be capable of inhibiting both cap- and viral internal ribosome entry site (IRES)-dependent translation initiation [50]. However, whether gemin5 actually binds and regulates translation of endogenous cellular or viral mRNAs in vivo is still an open question deserving further inquiry.

Translational repression mediated by microRNAs (miRNAs) represents one scenario where mRNAs may be transiently remodeled into silenced particles prior to decay or resumption of active translation. Though mechanisms of miRNA function remain obscure [51], [52], multiple studies have implicated the m^7^G cap structure as a cis-acting element that confers susceptibility to repression by miRNAs [53], [54], [55], [56]. Indeed, the miRNA-associated protein Ago2 was reported to repress mRNA translation through direct interaction with the 5′ cap [57], although this claim has been recently questioned [58]. Given its association with gemin3 and gemin4 [21], both of which appear to participate in complexes with Ago2 [34], [35], and its m^7^G-binding capability reported here, a possible role for gemin5 in miRNA-mediated silencing deserves examination.

## Materials and Methods

### Cap-affinity chromatography and western blotting

HeLa and 293T cell lysates were prepared using polysome lysis buffer (PLB) as described [59]. Cap-binding reactions were performed at 4°C with one mg protein and 15 µl m^7^GTP-sepharose or sepharose-4B (GE Healthcare) in NT2 buffer [50 mM Tris-HCl (pH 7.4), 150 mM NaCl, 1 mM MgCl_2_, 0.05% NP40] for one hour. Binding reactions were performed in the presence or absence or 0.1 mM cap analogs (GpppG or m^7^GpppG). Sepharose beads were washed 3 times with 1 ml NT2 buffer and then resuspended in sample buffer for SDS-PAGE. Antibodies to eIF4E (Abcam), PABP [60], gemin5 (BD Biosciences), gemin4 (Santa Cruz), gemin3 (Abcam), FLAG-tag (Sigma), and myc-tag (Sigma) were used for western blotting. Antibody to eIF4G was produced at the Duke University antibody production facility. For preparative purification of cap-binding complexes the reaction described above was scaled up 4-fold and proteins detected by coomassie staining were analyzed at the University of Massachusetts Medical School Proteomics Facility (Worcester, MA).

### Immunoprecipitations and UV crosslinking

Buffers used for lysate preparation (PLB) and binding reactions (NT2) were identical to those used in cap-binding assays. IP reactions were incubated one hour and contained 40 µl protein G-sepharose (GE Health Sciences) bound to 10 µg antibody and 2 mg protein lysate. Washed immunoprecipitates were resuspended in either sample buffer for SDS-PAGE, Trizol reagent (Invitrogen) for RNA extraction, or NT2 buffer with 0.1 mg/ml FLAG peptide (Sigma) for elution of FLAG-tagged proteins. For UV-crosslinking experiments, RNA containing the *Xenopus* elongation factor 1α gene was in vitro transcribed (Megascript, Ambion) and capped using vaccinia virus guanylyltransferase and [α-^32^P]-GTP according to the manufacturer's protocol (Ambion). Immunoprecipitated FLAG-tagged gemin5 and gemin5(Δ25) were concentrated using Microcon filter devices (Millipore). Crosslinking reactions (20 µl) contained 60 ng labeled RNA, 40 ng immunopurified protein or 120 µg cytoplasmic lysate, 50 mM Tris-HCl (pH 7.4), 150 mM NaCl, and 1 mM MgCl_2_. Reactions were incubated 10 minutes at RT and then irradiated 20 minutes on ice with a UV Stratalinker (Stratagene). After crosslinking, reactions were treated with 1 µl RNase cocktail (Ambion) for 10 minutes at RT and then subjected to SDS-PAGE.

### Cloning of cDNA constructs and transfections

Gemin5 cDNA clone was obtained from ATCC (RefSeq: NM_015465) and the coding region was inserted into pcDNA 3.1 (Invitrogen) using *Kpn*I and *Not*I. This clone was modified with a C-terminal FLAG tag by PCR. The N- and C-terminal deletion constructs were derived in a similar fashion. Point mutations were generated by overlap fusion PCR [61] and standard cloning techniques. The eIF4E clone was established by RT-PCR amplification of HeLa total RNA and the PCR fragment was inserted into pcDNA 3.1 containing a myc-tag [62]. N-terminally FLAG-tagged Ago2 in pcDNA 3.1 was derived by standard methods from a cDNA clone (ATCC; RefSeq:NM_012154). All clones were sequenced to ensure correctness. Transfection of expression plasmids for IP or cap-pull down experiments was performed with 293T cells grown on 10 cm dishes. Cells were transfected with 10 µg DNA and 40 µl lipofectamine 2000 (Invitrogen). Twenty-four hours later cells were harvested and lysed as described above.

### Structural modeling

The PHYRE program [37] was used to model the WD repeat domain structure of gemin5 as described previously [36]. PHYRE analysis predicted actin-interacting protein 1 (AIP-1; Q11176) as a factor with significant structural homology to the WD repeat region of gemin5. The solved structure of AIP-1 [38] was used to model positions of gemin5 amino acids W286, F304 and F338 as well as the N-terminus. Structures were visualized using Cn3D [63].

### Quantitative RT-PCR (RT-qPCR)

One step RT-qPCR reactions were performed on co-precipitating RNA from IP reactions using SYBR green reaction mix (Roche) and the following primers specific for target RNAs: (U1 snRNA-forward) 5′-GGGAGATACCATGATCACGAAGGT-3′; (U1 snRNA-reverse) 5′-ATGCAGTCGAGTTTCCCACA-3′; (U6 snRNA-forward) 5′-CTCGCTTCGGCAGCACATATACTA-3′; (U6 snRNA-reverse) 5′-ACGAATTTGCGTGTCATCCTTGCG-3′. Real-time PCR was conducted using a Roche Light Cycler. Reactions were performed in triplicate on at least two separate experiments for each gemin5 variant and the ΔΔCt method was used to calculate fold changes in positive versus negative IPs. A representative experiment is shown in figure 7. Statistical analysis was performed as described [64].
